# Clinical and Biological Relevance of Genomic Heterogeneity in Chronic Lymphocytic Leukemia

**DOI:** 10.1371/journal.pone.0057356

**Published:** 2013-02-28

**Authors:** Daphne R. Friedman, Joseph E. Lucas, J. Brice Weinberg

**Affiliations:** 1 Department of Medicine, Duke University, Durham, North Carolina, United States of America; 2 Institute for Genome Sciences and Policy, Duke University, Durham, North Carolina, United States of America; 3 Department of Medicine, Duke University and Durham VA Medical Centers, Durham, North Carolina, United States of America; University of North Carolina at Chapel Hill, United States of America

## Abstract

**Background:**

Chronic lymphocytic leukemia (CLL) is typically regarded as an indolent B-cell malignancy. However, there is wide variability with regards to need for therapy, time to progressive disease, and treatment response. This clinical variability is due, in part, to biological heterogeneity between individual patients’ leukemias. While much has been learned about this biological variation using genomic approaches, it is unclear whether such efforts have sufficiently evaluated biological and clinical heterogeneity in CLL.

**Methods:**

To study the extent of genomic variability in CLL and the biological and clinical attributes of genomic classification in CLL, we evaluated 893 unique CLL samples from fifteen publicly available gene expression profiling datasets. We used unsupervised approaches to divide the data into subgroups, evaluated the biological pathways and genetic aberrations that were associated with the subgroups, and compared prognostic and clinical outcome data between the subgroups.

**Results:**

Using an unsupervised approach, we determined that approximately 600 CLL samples are needed to define the spectrum of diversity in CLL genomic expression. We identified seven genomically-defined CLL subgroups that have distinct biological properties, are associated with specific chromosomal deletions and amplifications, and have marked differences in molecular prognostic markers and clinical outcomes.

**Conclusions:**

Our results indicate that investigations focusing on small numbers of patient samples likely provide a biased outlook on CLL biology. These findings may have important implications in identifying patients who should be treated with specific targeted therapies, which could have efficacy against CLL cells that rely on specific biological pathways.

## Introduction

Chronic lymphocytic leukemia (CLL) is a generally indolent B-cell malignancy. However, since the time that clinical staging systems were developed [1], [2], it has been appreciated that there is clinical variability between CLL patients. Efforts to better characterize this variability led to the identification and extensive validation of numerous molecular prognostic markers [3], [4], [5], [6], [7]. Research in this field has highlighted the concept that molecular markers can link biology with clinical outcomes. For example, CD38 and ZAP70 are involved in surface receptor signaling, and patients with high expression of these markers have worse survival outcomes [8]. Thus, biologic heterogeneity, defined by these markers, appears to underlie clinical variability.

The use of gene expression profiling of CLL cells as an experimental approach has informed the understanding of CLL biology dramatically. Comparisons of genomic expression of CLL cells from patients grouped by clinical outcomes or by prognostic markers have led to the identification of numerous genes or gene signatures associated with these phenotypes [4], [9], [10], [11], [12], [13], [14], [15], [16], [17]. Our previous work identified genomic signatures that were associated with prognosis and response to treatment in limited numbers of CLL patients [12]. Further research has studied identified genes, such as ZAP70, to identify their biological and cellular significance [18], [19], [20], [21]. Genomic approaches have also informed research on the CLL microenvironment, for example dissecting biological differences of CLL cells that reside in different anatomic niches [22].

Despite these contributions, it is unclear whether the gene expression profiling approach used to date is sufficient. The marked clinical and biological heterogeneity of CLL and limited numbers of subjects in individual studies may make it difficult to fully and accurately assess genomic heterogeneity in CLL. To address this methodically and objectively, we evaluated publicly available CLL gene expression datasets in concert in order to assess the number of samples required to capture genomic heterogeneity in CLL and to determine if CLL subgroups defined by gene expression profiling have biological and clinical relevance.

## Methods

### Dataset Selection, Combination, and Normalization

The Gene Expression Omnibus (GEO) at the National Center for Biotechnology Information (NCBI) website was queried using terms including lymphoma, B-cell lymphoma, CLL, chronic lymphocytic leukemia, small lymphocytic lymphoma, and SLL. Datasets were then filtered to include only *Homo sapiens* and Affymetrix U133 generation gene expression arrays. Thereafter, datasets were manually sorted to remove duplicate data files (between datasets) or data files that represented culture experiments. Of the fifteen identified datasets, one publicly available dataset was from our previous research at the Duke University and Durham VA Medical Centers [12], [22], [23], [24], [25], [26], [27], [28], [29], [30], [31], [32], [33], [34].

Gene expression data (CEL) files from selected datasets were downloaded from the GEO website. CEL files were normalized using RMA and MAS5 normalization methods using the affy package in Bioconductor [35]. Thereafter, normalized datasets were filtered to common probes between the three chips (22,277 probes). The datasets were combined and normalized for batch effect with the Bayesian Factor Regression Method (BFRM), using the BFRM normalize module on the Duke license of GenePattern [36]. Batch effect was assessed prior to and after normalization using principal component analysis.

### Creation of Subgroups

We identified subgroups of the combined RMA normalized dataset by performing Consensus Clustering [37] with unsupervised hierarchical clustering (Euclidean distance and Ward agglomerative method) using the R package ConsensusClusterPlus. The dataset was initially filtered to the 10% of probes with the highest standard deviation across all samples (2222 probes). Within the Consensus Clustering algorithm, 90% of the samples were resampled in each of the 50 total iterations. We defined the number of subgroups (seven subgroups) based on the point on the delta area plot at which there was minimal relative decrease in the consensus cumulative distribution function (CDF). Thereafter, samples were assigned subgroup membership by performing unsupervised hierarchical clustering on the complete filtered dataset using the same clustering settings.

### Sub-sampling of the Combined Dataset

The combined dataset was reordered randomly. Subsequently, sub-datasets of the combined dataset (comprised of first 50 samples, the first 100 samples, and so on in multiples of 50 samples, through the entire dataset) were defined. The probes in these sub-datasets were filtered to approximately the top 10% with the highest standard deviation (2222 probes). Thereafter, Consensus Clustering was performed on the sub-datasets using the settings outlined above. This process was iterated twenty-five times. CDF plots for the sub-datasets were compared to CDF plot for the entire dataset and scored for similarity in terms of area under the curve and slope of the curves. Statistical difference in score of the sub-datasets compared to the entire dataset was assessed using the Fisher’s exact test.

### Gene and Pathway Annotation

Assessment of gene and genomic pathway annotation was performed using Gene Set Enrichment Analysis (GSEA) [38] and genomic signatures of oncogenic pathway deregulation (ScoreSignature) [36], [39]. For GSEA analysis, RMA normalized data were used; for ScoreSignature analysis, both RMA and MAS5 normalized data were used. In GSEA, all probes in the combined gene expression dataset were used, and samples in each subgroup were compared to the remaining samples. Since the purpose of this analysis was exploratory, we considered any gene set with a nominal p value of less than 0.01 to be significant. Genomic signatures of oncogenic pathway activation were evaluated in the combined dataset using the Duke GenePattern license. Significant differences in pathway scores between subgroups were evaluated using the Kruskal-Wallis rank sum test.

### Single Nucleotide Polymorphism (SNP) Analysis

GSE16746 contained 60 samples that had been arrayed with Affymetrix U133A gene expression array and with Affymetrix 250K Nsp SNP Array [28]. The CNAT processed log2 copy number data which were posted to the GEO website were downloaded. Thereafter, using the DNAcopy package from bioconductor, circular binary segmentation was used to identify change-points at which the underlying DNA copy number was altered [40], [41]. This identified regions of SNPs that were linked together and had normal copy number or copy number variation (amplifications or deletions). With a value of 2 representing normal copy number, a value of 2.2 or greater was considered to be an amplification, and a value of 1.8 or below was considered to be a deletion. Copy number variation in chromosome X was not evaluated, since sex of the CLL patients was not known. We focused on copy number variation found in two or more CLL samples.

The samples in GSE16746 were assigned into subgroups based on their branch membership in unsupervised hierarchical clustering. The frequency of each copy number variation within each subgroup was compared to the expected frequency using the Pearson’s Chi-squared test, and regions of copy number variation with a p-value of less than 0.05 was considered significant. Insufficient total number of samples per group precluded performing multiple testing corrections.

### Clinical Variable Analysis

Molecular prognostic marker and outcome data were gathered for each sample included in the analysis, if available. In most cases, the data were available on GEO or in supplemental tables in the publications, but in some cases we retrieved the information from the corresponding author directly.

Samples in the combined dataset were separated based on subgroup. Significant differences in available molecular prognostic markers between subgroups were assessed using Pearson’s Chi-squared test. For datasets with clinical treatment response data (GSE15490 and GSE10137) [12], [25], the rates of complete response, partial response, stable disease, and progressive disease were compared between samples in different subgroups using Pearson’s Chi-squared test. For the dataset with clinical outcome data (GSE10138) [12], samples were divided by genomic subgroup, and Kaplan-Meier analysis was performed to assess overall survival divided on subgroup. The log-rank test was used to statistically compare clinical outcomes between groups. A p-value of less than 0.05 was considered to be significant.

### Statistics and Computational Analysis Methods

Statistical analyses, raw gene expression normalization, Consensus Clustering, and circular binary segmentation were performed using R. Genomic pathway analyses were performed using Gene Set Enrichment Analysis (java applet from the Broad Institute, version 2). We used the Duke University GenePattern server (https://genepattern.genome.duke.edu/gp/pages/login.jsf) [42] to assess genomic signatures of oncogenic pathway activation and perform BFRM normalization. Code used to perform the analyses in R can be found in the Text S1.

## Results

### Dataset Characteristics and Processing

From a query of the GEO database for CLL-containing datasets, we identified fifteen datasets that contained 893 unique CLL sample data files (Table 1). The number of data files within each dataset ranged from eleven to 448. Associated CLL molecular prognostic markers (interphase cytogenetics, CD38 and ZAP70 expression, and IgV_H_ mutation status) were available for many, but not all, data files.

**Table 1:** Publicly available datasets, with available molecular prognostic factors.

Dataset	Batch (Figure 1)	Reference Number	Number of Samples	Interphase Cytogenetics	CD38	ZAP70	IgV_H_ Mutation Status
GSE6691	1	30	11	NA: 11	NA: 11	NA: 11	NA: 11
GSE9250	2	27	20	13qdel: 10	NA: 20	Neg: 14	M: 15
				Normal: 10		Pos: 6	UM: 5
GSE9992	15	33	60	17qdel: 7	NA: 60	NA: 60	M: 24
				NA: 53			UM: 36
GSE10137	3	12	40	NA: 40	Neg: 26	Neg: 21	M: 15
					Pos: 14	Pos: 15	UM: 25
						NA: 4	
GSE10138	4	12	68	13qdel: 18	Neg: 51	Neg: 18	M: 39
				Normal: 14	Pos: 14	Pos: 44	UM: 26
				Tri12∶9		NA: 6	NA: 3
				11qdel: 4			
				17pdel: 5			
				NA: 18			
GSE12734	5	24	14	13qdel: 3	Neg: 4	Neg: 7	M: 8
				Normal: 3	Pos: 10	Pos: 7	UM: 6
				Tri12∶2			
				11qdel: 1			
				17pdel: 1			
				6qdel: 4			
GSE13159	10	29	448	NA: 448	NA: 448	NA: 448	NA: 448
GSE15490	12	25	20	13qdel: 5	NA: 20	NA: 20	M: 7
				Normal: 3			UM: 11
				Tri12∶4			NA: 2
				11qdel: 5			
				17pdel: 2			
				NA: 1			
GSE15777	14	34	22	NA: 22	Neg: 13	Neg: 14	M: 16
					Pos: 5	Pos: 6	UM: 4
					NA: 4	NA: 2	NA: 2
GSE15913	6	31	20	13qdel: 6	Neg: 14	Neg: 8	M: 1
				Normal: 5	Pos: 5	Pos: 10	UM: 19
				Tri12∶1	NA: 1	NA: 2	
				11qdel: 5			
				17pdel: 3			
GSE16455	7	32	17	NA: 17	NA: 17	NA: 17	NA: 17
GSE16746	8	28	60	13qdel: 17	Neg: 31	Neg: 39	M: 23
				Normal: 17	Pos: 29	Pos: 21	UM: 37
				Tri12∶12			
				11qdel: 7			
				17pdel: 7			
GSE21029	9	22	62	13qdel: 22	Neg: 27	Neg: 21	M: 26
				Normal: 6	Pos: 35	Pos: 41	UM: 36
				Tri12∶15			
				11qdel: 12			
				17pdel: 5			
				NA: 2			
GSE26526	11	26	19	13qdel: 2	NA: 19	Neg: 5	M: 4
				Normal: 6		Pos: 14	UM: 15
				Tri12∶1			
				11qdel: 10			
GSE26725	13	23	12	13qdel: 2	Neg: 5	Neg: 4	M: 2
				Normal: 1	Pos: 7	Pos: 7	UM: 9
				Tri12∶1		NA: 1	NA: 1
				11qdel: 6			
				17pdel: 2			

Fifteen publicly available datasets were obtained from GEO that had unique gene expression profiling files representing CLL. The number of CLL samples per dataset ranged from 11 to 448. Molecular prognostic markers that correspond to the gene expression profiling data were available for a majority of the total samples, but the prognostic markers were not evenly spread between the different datasets, largely owing to experimental design of each dataset. NA signifies data not available, Neg represents negative, Pos represents positive, M represents IgV_H_ mutated, and UM represents IgV_H_ unmutated.

**Figure 1:**
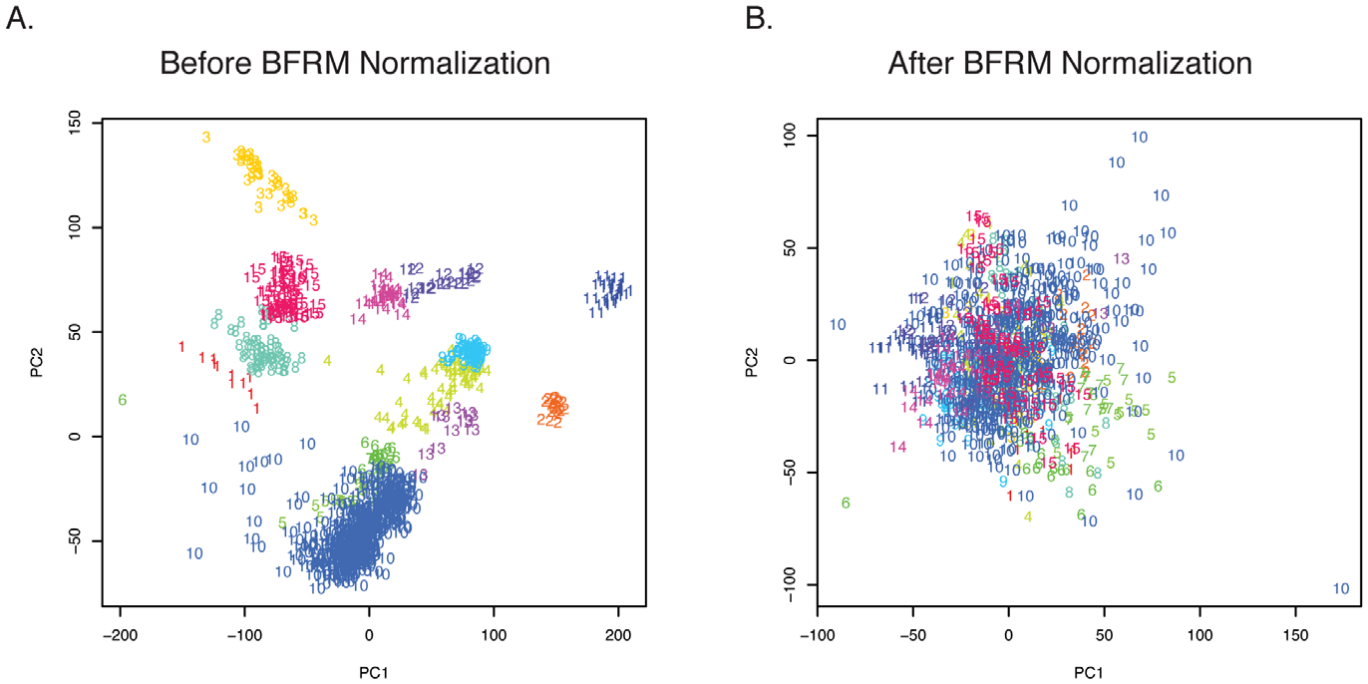
CLL gene expression data files from the fifteen individual datasets were evaluated by principal component analysis (PCA). A) PCA prior to Bayesian Factor Regression Modeling (BFRM) normalization was performed, and the first principal component (PC) is plotted against the second PC. Numbers represent dataset order found in Table 1. CLL samples from each dataset cluster together. B) PCA following BFRM normalization was performed, and the first PC is plotted against the second PC. Samples retain the same numbering as in Figure 1A. CLL samples now cluster together in one cloud.

After downloading datasets from GEO and normalizing the files from individual datasets, we combined the datasets, filtering the data to include common probes. The combined dataset was further normalized with the Bayesian Factor Regression Method (BFRM) to reduce batch effect. We evaluated the efficacy of this normalization process using principal component analysis of the combined dataset prior to and after normalization. Prior to BFRM normalization, samples were grouped with other samples from the same dataset (Figure 1A). However, after BFRM normalization, samples were spread evenly with samples from other datasets (Figure 1B), demonstrating that batch effect has been reduced dramatically.

### Evaluating the Extent of Genomic Complexity

**Figure 2:**
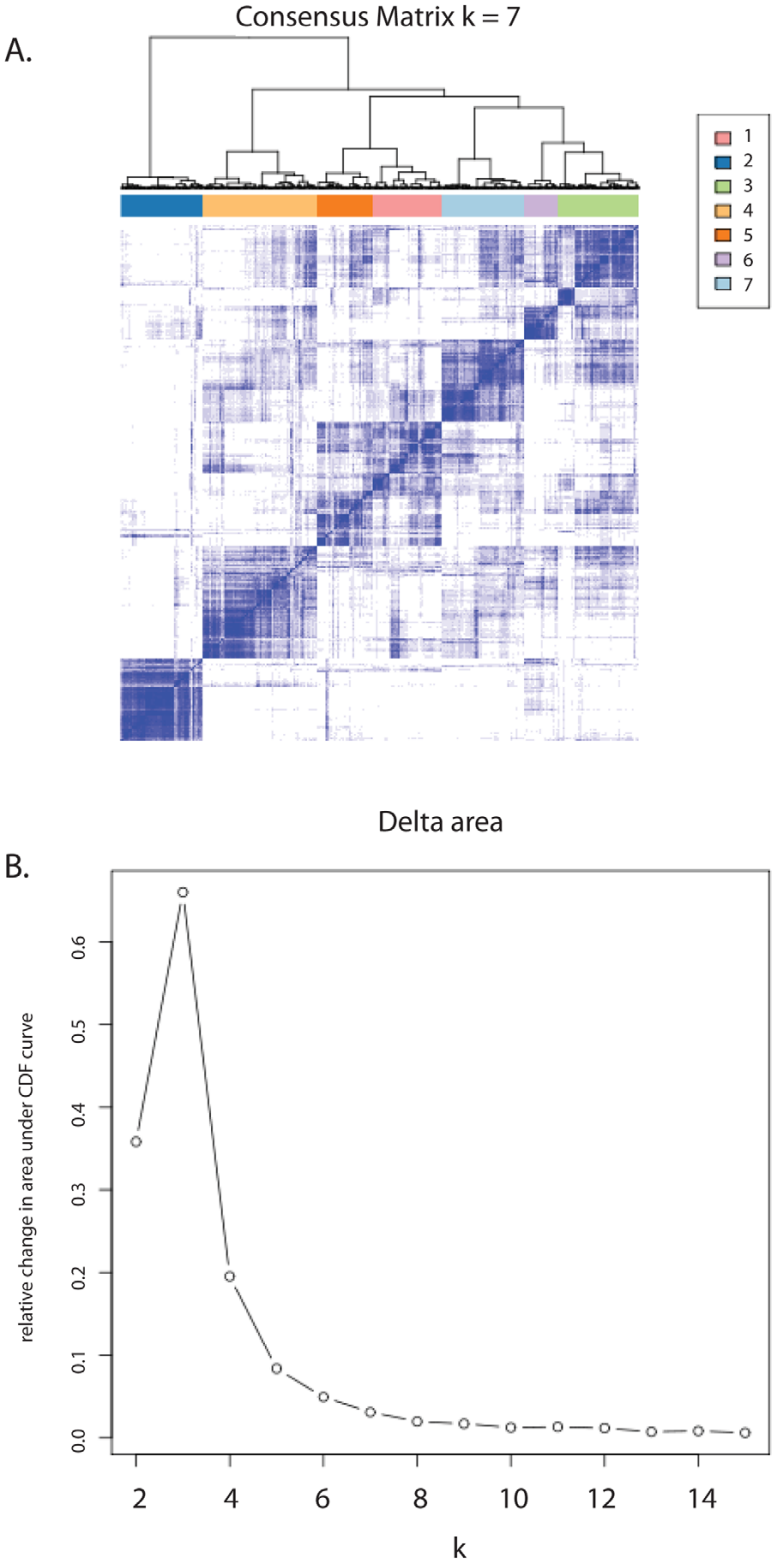
Results of Consensus Clustering to define the smallest number of subgroups that define genomic variation within the entire combined dataset. A) A heatmap of the consensus matrix, displaying samples with high consensus (blue) are grouped together, compared to those with low consensus (white). B) The delta area plot shows a negligible increase in area under the Consensus cumulative distribution function in more than seven subgroups. Thus, dividing the data into more subgroups does not improve sample classification.

To assess the extent of genomic heterogeneity in CLL, we evaluated the combined dataset with unsupervised hierarchical clustering using the Consensus Clustering algorithm. As seen in Figure 2, this approach identifies seven subgroups that group together based on consensus. Further dividing of the entire dataset into a larger number of subgroups has minimal improvement in classification, as demonstrated by the minimal change in the Consensus cumulative distribution function (CDF) beyond seven subgroups.

**Figure 3:**
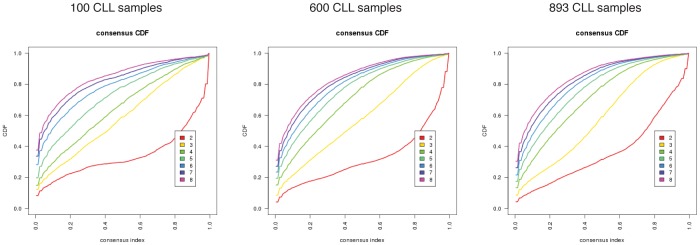
Representative examples of Consensus Cumulative Distribution Function (CDF) plots for the entire dataset (right) and randomly selected sub-datasets of 100 and 600 CLL samples (left and middle, respectively). By evaluating area under the curve and slope of the curves, it is appreciated that CDF plots of Consensus Clustering of sub-datasets the include 600 CLL samples are similar to the CDF plot of the entire dataset containing 893 CLL samples. However, CDF plots obtained upon using smaller sub-datasets, for example comprised of 100 CLL samples, is not similar to the CDF plot of the entire dataset.

Before assessing the biological and clinical relevance of genomically-defined CLL subgroups, it was important to determine if the number of samples in the combined dataset were sufficient to evaluate genomic heterogeneity in CLL. Assuming there is no bias in the availability of genomic data, we would expect that increasing the number of samples in the combined dataset would cease to increase the number of subgroups once maximum genomic heterogeneity has been reached. Therefore, we evaluated the combined dataset in an iterative fashion to determine if a smaller number of CLL samples could be used to obtain the same subgroups as the entire combined dataset. To do so, we used the Consensus Clustering algorithm to evaluate the CDF of two to eight subgroups on increasing numbers of randomly selecting samples from within the entire dataset. This process was repeated 25 times. CDF plots of sub-datasets were compared to the CDF plot of the entire dataset. The CDF plots for sub-datasets of 50 to 550 samples were different than the CDF plot for the entire dataset (p<0.0001, Fisher’s Exact Test), whereas 600 to 850 samples were not statistically different than the CDF plot for the entire dataset (p>0.05, Fisher’s Exact Test). Representative plots are displayed in Figure 3. Thus, approximately 600 or more CLL samples are required to evaluate genomic complexity in CLL as a whole.

### Genomically-defined CLL Subgroups and their Biological Relevance

As described above, we identified seven CLL subgroups by performing unsupervised hierarchical clustering on the entire combined dataset. We sought to determine if these genomically-defined CLL subgroups have biological relevance, using gene and genomic pathway annotation tools such as Gene Set Enrichment Analysis (GSEA) and genomic signatures of oncogenic pathway activation. As seen in Table 2, the seven subgroups differed in terms of pathways identified using GSEA. Biological processes known to be important in CLL were identified (such as B-cell receptor signaling and the NF-κB pathway). However, we noted other pathways not traditionally focused on in the study of CLL biology. For example, RNA processing and interferon pathways were identified as associated with certain CLL subgroups.

**Table 2:** Genomically-defined CLL subgroups with biological annotation.

Group	Number of Samples	Enriched pathways identified in GSEA
1	88	RNA processing, TNFα and MAPK pathways, proteosome and ubiquitination
2	120	Cytokine/Interferon receptor signaling, cell motility and adhesion, RAS pathway
3	225	Hematopoietic progenitor cell, amino acid metabolism
4	90	Suppression of TNFα, TGF-β, and MAPK pathway activity
5	168	B-cell receptor signaling
6	32	Interferon pathway, NOTCH signaling, MYC pathway
7	170	Suppression of MYC pathway and TACI receptor signaling

Unsupervised hierarchical clustering of the combined and normalized gene expression profiling dataset defined seven CLL subgroups. The number of CLL samples per subgroup ranged from 32 to 225. Gene Set Enrichment Analysis (GSEA) was used to evaluate biological pathways that distinguished each subgroup from the others.

**Figure 4:**
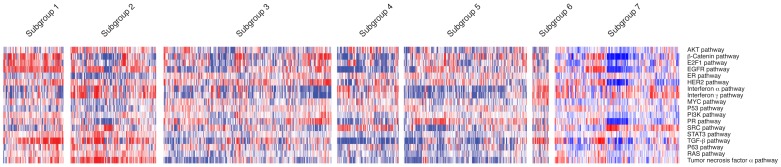
A heatmap of oncogenic pathway signature predictions, with CLL samples grouped by genomically-defined subgroups on the x-axis, and signatures on the y-axis. Red denotes high signature prediction, and blue denotes low signature prediction, with prediction scores scaled by row. This demonstrates that subgroups have distinct patterns of oncogenic pathway activity, which confirm results obtained from GSEA analysis.

The annotations revealed by GSEA were consistent with our analysis using gene expression signatures that measure oncogenic and cell signaling pathways. These signatures were developed from experimental perturbations of pathways and provide a quantitative estimate of the state of the cellular pathway in a given sample [36], [39]. As displayed in Figure 4, the predictions of pathway activity using these signatures revealed distinctions between the CLL subgroups. For example, subgroups one and two were found to have high TNFα/NF-κB pathway activity and subgroup four was found to have low activity, consistent with the analysis from GSEA. Further, subgroups two and six exhibited elevated interferon alpha and gamma pathway activity, again consistent with the annotations obtained by GSEA. These analyses underscore that subgroups defined by raw gene expression data have differences in underlying biology and pathway activation.

Since copy number variations (CNV) in the CLL leukemia cells could contribute to alterations in gene expression, we assessed the extent to which amplifications and deletions were associated with specific genomically-defined CLL subgroups. We could assess this using one dataset (GSE16746) in which sixty CLL samples were simultaneously evaluated using gene expression profiling and single nucleotide polymorphism (SNP) arrays. The sixty samples were subdivided based on their genomic subgroup, and CNVs were evaluated for statistically significant enrichment within subgroups. CNVs that represented commonly tested cytogenetic aberrations from fluorescent *in situ* hybridization (FISH) were not compared in this analysis since this information was captured by otherwise obtained FISH data. Table 3 outlines the four genetic regions significantly enriched within particular subgroups. Within these regions of amplification or deletion, genes related to processes known to have biological or clinical relevance were identified, including those involved in lipid and lipid-related hormone signaling (LRP5L, LEPR, LPIN1, APOB) and tumor necrosis factor and NF-κB pathways (ADAM17, REL). Thus, an unsupervised method of grouping CLL samples identifies and enriches for CNVs. These CNVs would be overlooked when considering CLL as one entity. These results reinforce the concept that these CLL subgroups have genetic and biologic significance.

**Table 3:** Single nucleotide polymorphism deletions and amplifications that are statistically enriched in genomically-defined CLL subgroups.

Chromosome	Genes	Variation	Enriched in Subgroups	Reduced in Subgroups	P value
22q11.23	LRP5L	Deletion	3	1, 7	0.037
	CRYBB2				
	CRYBB2P1				
	LOC91353				
1p31.3	LEPR	Amplification	4	1, 7	0.0004
2p25.3–p22.2	229 genes including:	Amplification	1, 2, 5	3, 7	0.012
	ADAM17				
	E2F6				
	LPIN1				
	NT5C1B				
	RHOB				
	APOB				
	ALK				
	PPP1CB				
	SOCS5				
	MSH2				
	BCL11A				
	REL				
	XPO1				

An evaluation of copy number variations in CLL lymphocytes revealed two regions of amplification and one region of deletion that are significantly associated with certain subgroups. The regions were identified based on Affymetrix annotation, and was verified with the University of California Santa Cruz genomic browser, NCBI135/hg17 genome assembly. A full list of the genes contained in amplification region on chromosome two is found in Table S1. P values were calculated using the Pearson’s Chi-squared test.

### Genomically-defined CLL Subgroups and their Clinical Relevance

Molecular prognostic markers, including IgV_H_ mutation status, ZAP70 and CD38 expression, and interphase cytogenetic aberrations, identify subgroups of CLL patients with varying clinical outcomes. We hypothesized that genomically-defined CLL subgroups would be significantly associated with these prognostic markers and would have different clinical outcomes. We assessed the extent to which high-risk CLL prognostic markers are associated with CLL subgroups, and found a significantly different distribution of these markers in different subgroups (Table 4). Each prognostic marker differed significantly between the subgroups (p<0.0001 for CD38 status, ZAP70 status, IgV_H_ mutation status, and interphase cytogenetics, Pearson Chi-squared test). Comparing the different subgroups, the percent of samples that were IgV_H_ unmutated ranged between 25% and 82%, the percentage of ZAP70 positive samples ranged between 0% and 83%, the percentage of CD38 positive samples ranged between 18% and 75%, and the percentage of 17p or 11q deletion samples ranged between 7% and 100%. It is notable that CLL samples with particular prognostic markers are not exclusively found within specific genomically-defined CLL subgroups. This likely reflects a level of heterogeneity not fully captured by these commonly used markers. Since the molecular prognostic markers are associated with clinical outcome endpoints, differences in frequency of these markers within genomically-defined CLL subgroups could indicate that the differences between these subgroups underlie an important part of the observed clinical variation observed in CLL.

**Table 4:** High-risk molecular prognostic markers found in each genomically-defined CLL subgroup.

Group	% (n) 17pdel or 11qdel (FISH)	% (n) CD38 Positive	% (n) ZAP70 Positive	% (n) IgVH Unmutated
1	19% (6/31)	49% (18/37)	58% (21/36)	60% (33/55)
2	100% (3/3)	75% (3/4)	67% (2/3)	80% (4/5)
3	37% (38/101)	61% (46/76)	83% (77/93)	82% (96/117)
4	29% (9/41)	18% (6/34)	64% (21/33)	64% (24/39)
5	37% (15/41)	26% (14/54)	36% (20/56)	25% (17/68)
6	7% (1/14)	38% (6/16)	0% (0/16)	44% (8/18)
7	17% (10/58)	38% (26/69)	35% (30/85)	43% (46/107)

An evaluation of molecular prognostic markers found in the genomically-defined CLL subgroups identifies significantly different levels of these markers between the subgroups (p<0.0001, Pearson’s Chi-squared test for each prognostic marker). Results are reported as percentage of samples within a group with each high-risk prognostic marker, calculated as number with the prognostic marker divided by the total within the subgroup with data available.

**Figure 5:**
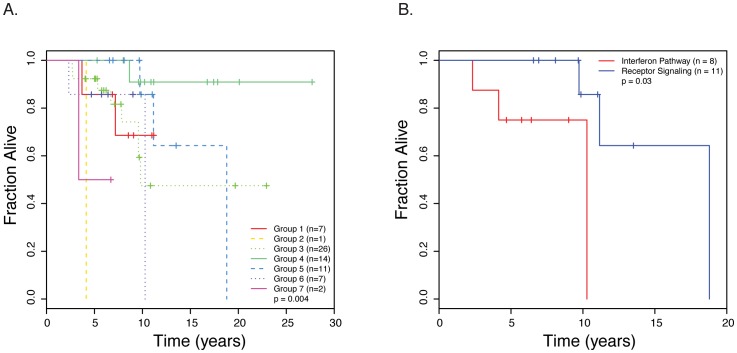
Kaplan-Meier analysis of time from diagnosis to treatment in sixty-eight CLL patient samples, grouped by genomically-defined subgroup. A) A significant difference in overall survival was observed between CLL subgroups (p  = 0.004). B) CLL patients in “Interferon Pathway” subgroups had inferior overall survival compared to CLL patients in the “Receptor Signaling” subgroup (p  = 0.03). Significance was assessed using the log-rank test.

To assess the relationship between the CLL subgroups and clinical outcomes further, we evaluated overall survival in 68 CLL samples from our institution evaluated previously (GSE10138) [12]. When we updated our clinical outcomes data, we found that patients grouped based on the genomically-defined subgroups had significantly different overall survival (Figure 5A, p  = 0.004, log-rank test). Of the molecular prognostic markers, CD38 and FISH results were significantly associated with overall survival in this cohort (p  = 0.047 and 0.01 respectively, log-rank test), whereas IgV_H_ and ZAP70 status were not. We then assessed the extent to which subgroups with particular gene and pathway annotations had disparate clinical outcomes. As seen in Figure 5B, we found that CLL patients that fell into subgroups with interferon pathway annotations (subgroups two and six) had significantly worse outcomes than patients with samples that fell into the subgroup with B-cell receptor signaling annotations (subgroup five). These pathway annotations were evaluated because B-cell receptor signaling is a known important cellular pathway in CLL, while the interferon pathway and inflammation has not been traditionally studied with regards to CLL biology.

We assessed the extent to which the genomically-defined subgroups could be used not only as prognostic markers, but as predictive markers of therapy response. When we evaluated two datasets that included response to treatment data (GSE10137 and GSE15490), we found no significant correlation between CLL subgroup and response to chemo-immunotherapy regimens (p>0.05, Pearson’s Chi-squared test). In sum, these results indicate that CLL subgroups, defined in an unsupervised manner with raw gene expression data, are based on biological processes and have prognostic relevance in terms of overall survival.

## Discussion

Clinical variability in CLL is widely appreciated but poorly understood. By pooling and evaluating publicly available gene expression profiling data using unsupervised methods, we defined subgroups of CLL that have unique biological and clinical differences. This evaluation of genomic subgroups is not meant to replace currently used clinical prognostic markers. However, it is meant to demonstrate that CLL is much more heterogeneous (genetically, biologically, and clinically) than can be accounted for using current prognostic markers.

Our results could have been affected by potential confounders such as bias in publicly available datasets and the lack of associated prognostic and clinical outcome data for all samples. The agreement in our evaluation of the data using two methods of assessing biological significance suggests that our approach and methods are valid. Additionally, the concordant results in two datasets with regards to response to therapy stratified by genomic subgroup also suggests that these confounders are likely not influencing our analysis. At the most, including additional data might increase the identified genomic heterogeneity in CLL as a whole.

Our evaluation of the extent of genomic heterogeneity in CLL demonstrates that approximately 600 or more unique samples are required to divide CLL into subgroups with the identified clinical and biological relevance. Because this number of total samples may be too great for typical clinical or translational research, a focus on specific subgroups would enrich for genetic or biologic backgrounds that could have particular relevance.

Genomic research in CLL is moving towards next-generation whole exome and whole genome sequencing approaches. Several studies using next-generation sequencing approaches identified SNPs in CLL cells with clinical relevance in independent large cohorts of CLL patients. For example, NOTCH1 mutation was found in 4–12% of CLL patients [43], [44], [45]. NOTCH1 mutations, which cause constitutively active NOTCH signaling, are associated with poor prognosis and are more prevalent in advanced CLL [45]. We found that activation of the NOTCH pathway was significantly associated with subgroup six, which constitutes approximately 4% of the combined dataset, but was not associated with a significantly reduced overall survival compared to other subgroups. Regarding the XPO1 mutation, which was found in 2.4% of CLL patients in one study [44], we noted that amplification of XPO1 was enriched in subgroups one, two, and five. Lastly, mutations in SF3B1, a component of the mRNA splicing complex were found in 15% of CLL patients from one recent study, and was associated with the 11q deletion and poor clinical outcome [43]. We found that subgroup one was defined by activity of mRNA processing and splicing (including enrichment of other splicing factor 3B subunit genes). While our evaluation of clinical impact of these particular genes in the combined dataset is limited by incomplete clinical data, our validation of these recently identified polymorphisms using gene expression data suggests that the different genomic approaches can complement each other. In addition, this work also implies that future efforts to focus next-generation sequencing on more homogeneous CLL populations (defined by gene expression profiling) could enrich for particular genomic aberrations and could reduce the total number of patients needed for such studies.

Research using gene expression profiling has informed laboratory-based and clinical investigations and clinical practice in CLL. In part because of the resulting improved understanding of CLL biology, therapies targeting important pathways in CLL, such as B-cell receptor signaling and downstream second messenger systems, are being developed and clinically evaluated. Within the context of our results, these targeted therapies may have selective efficacy in certain genomically-defined subgroups. The assessment of patient responses to such targeted therapies, stratified by gene expression data, would validate the notion of using targeted therapies for subsets of CLL patients defined by leukemia biology.

Our work also identified CLL subgroups that are associated with biological processes that have not been extensively studied in the context of CLL biology, for example RNA processing and interferon pathways/inflammation. Importantly, we found that CLL samples, grouped by biological annotation, are associated with differences in overall survival in CLL patients. These results may lead to laboratory-based investigation to understand the functions of these pathways in CLL, validation within other patient cohorts, and potentially the development of additional novel targeted therapies.

In conclusion, we have found that the evaluation of genomic data from a large number of CLL patients allows us to identify heterogeneity within CLL and to learn more about the genomic, biologic, and clinical differences that span this malignancy. Dividing patients into distinct genomic groups could have implications for future research, for CLL prognosis, and for developing targeted therapeutics and associated biomarkers.

## Supporting Information

Table S1 **Full list of genes contained in the amplified region on chromosome two, enriched in subgroups 1, 2, and 5.**
(PDF)

Text S1 **Code used to perform the analyses in the manuscript, using the statistical environment R.**
(PDF)
