# Supplementary material for: Clinical and Biological Relevance of Genomic Heterogeneity in Chronic Lymphocytic Leukemia
Source: PLoS One. 2013 Feb 28;8(2):e57356. doi: 10.1371/journal.pone.0057356 (PMC3585365; doi:10.1371/journal.pone.0057356)
Supplement: Table S1 — Full list of genes contained in the amplified region on chromosome two, enriched in subgroups 1, 2, and 5. (PDF) [file pone.0057356.s001.pdf]

Supplementary Table 1

Full list of genes in amplified region 2p25.3-p15 in Table 3:

SH3YL1
ACP1
LOC285016
TMEM18
SNTG2
TPO
PXDN
MYT1L
TSSC1
TTC15
ADI1
RNASEH1
RPS7
COLEC11
ALLC
SOX11
RSAD2
RNF144
C2orf46
ID2
KIDINS220
MBOAT2
DDEF2
ADAM17
YWHAQ
TAF1B
GRHL1
KLF11
RRM2
CYS1
C2orf48
HPCAL1
ODC1
NOL10
ATP6V1C2
PDIA6
KCNF1
C2orf50
PQLC3
ROCK2
E2F6
GREB1
NTSR2
LPIN1
TRIB2
FAM84A
NAG
DDX1
FAM49A
VSNL1
FLJ40869
KCNS3
RDH14
NT5C1B
OSR1
TTC32
WDR35
LAPTM4A
PUM2
RHOB
HS1BP3
C2orf43
GDF7
APOB
UBXD4
FLJ30851
PFN4
ITSN2
NCOA1
ADCY3
CENPO
LOC391356
RBJ
DNMT3A
POMC
DTNB
ASXL2
RAB10
SELI
C2orf39
HADHA
OTOF
GPR113
CIB4
CENPA
KCNK3
C2orf18
DPYSL5
MAPRE3
AGBL5
EMILIN1
TCF23
SLC5A6
CAD
SLC30A3
TRIM54
GCKR
ZNF512
XAB1
SUPT7L
MRPL33
RBKS
BRE
FOSL2
PLB1
SPDYA
PPP1CB
CLIP4
FLJ34931
ALK
YPEL5
LBH
LYCAT
CAPN13
GALNT14
EHD3
XDH
SRD5A2
MEMO1
SPAST
HDPY-30
NLRC4
YIPF4
BIRC6
TTC27
LTBP1
RASGRP3
FAM98A
CRIM1
FEZ2
VIT
STRN
HEATR5B
CCDC75
EIF2AK2
PRKD3
QPCT
CDC42EP3
FAM82A
CYP1B1
C2orf58
ARL6IP2
HNRPLL
GALM
GEMIN6
SFRS7
DHX57
SOS1
CDKL4
MAP4K3
TMEM178
THUMPD2
SLC8A1
LOC91461
EML4
COX7A2L

KCNG3
MTA3
OXER1
HAAO
ZFP36L2
THADA
PLEKHH2
DYNC2LI1
ABCG5
LRPPRC
PPM1B
PREPL
SLC3A1
C2orf34
SIX2
SIX3
SRBD1
PRKCE
EPAS1
ATP6V1E2
CRIPT
RHOQ
PIGF
SOCS5
MCFD2
TTC7A
C2orf61
CALM1
CALM2
KCNK12
MSH2
FBXO11
MSH6
FOXN2
CCDC128
SALF
GTF2A1L
LHCGR
FSHR
NRXN1
CHAC2
ASB3
PSME4
ACYP2
TSPYL6
SPTBN1
FLJ40298
RTN4
FLJ31438
RPS27A
CCDC88A
MTIF2
CCDC104
SMEK2
EFEMP1
PNPT1
FANCL
VRK2
BCL11A
PAPOLG
REL
PEX13
FLJ32312
KIAA1841
AHSA2
USP34
XPO1
FLJ13305
COMMD1
B3GNT2
EHBP1
TMEM17
LOC51057
