# Supplementary material for: Clinical and Biological Relevance of Genomic Heterogeneity in Chronic Lymphocytic Leukemia
Source: PLoS One. 2013 Feb 28;8(2):e57356. doi: 10.1371/journal.pone.0057356 (PMC3585365; doi:10.1371/journal.pone.0057356)
Supplement: Text S1 — Code used to perform the analyses in the manuscript, using the statistical environment R. (PDF) [file pone.0057356.s002.pdf]

Supplementary Methods
Computational code used to perform statistical analyses using R.

Principal Component Analysis:
BFRM Normalized Data:

```
cll.rma <- read.delim("normalized_RMA.gct", skip = 2)
cll.rmadata <- cll.rma[,-c(1:2)]
rownames(cll.rmadata) <- cll.rma[,1]
fit.cll <- prcomp(t(cll.rmadata))
new.cll <- predict(fit.cll)

palette(rainbow(15))

pdf("PCA_PostNorm_CLL_111611.pdf")
plot(new.cll[,1], new.cll[,2], type = "n", xlab = "PC1", ylab = "PC2")
text(new.cll[,1], new.cll[,2], labels = cll.clin[,3], col = palette()[cll.clin[,3]])
dev.off()
```

Data prior to BFRM Normalization:

```
cll.prenorm <- read.delim("CLL_Batches12_v2_ComBat_Data.txt")
cll.clin <- read.delim("CLL_Batches12_Clin_Info_v2_BFRMdata.txt")
fit.cll2 <- prcomp(t(cll.prenorm))
new.cll2 <- predict(fit.cll2)

palette(rainbow(15))

pdf("PCA_PreNorm_CLL_111611.pdf")
plot(new.cll2[,1], new.cll2[,2], type = "n", xlab = "PC1", ylab = "PC2")
text(new.cll2[,1], new.cll2[,2], labels = cll.clin[,3], col = palette()[cll.clin[,3]])
dev.off()
```

Creation of Subgroups:

```
cll.sd <- apply(cll.rmadata, 1, sd)
cll.sd.ord10perc <- order(cll.sd, decreasing = T)[1:2222]
cll.sub10perc <- cll.rmadata[cll.sd.ord10perc,]

library(ConsensusClusterPlus)
results <- ConsensusClusterPlus(cll.sub10perc, maxK = 15, reps = 100, pItem = .9, pFeature = 1, title =
"ConsensusCluster_CLL_v4", clusterAlg = "hc", innerLinkage = "ward", finalLinkage = "ward", distance =
"euclidean", seed = 12345, plot = "png")

D <- as.dendrogram(hclust(dist(t(cll.sub10perc)), method = "ward"))
cut.D <- cut(D, h = 450)

cut.D$lower
[[1]]
'dendrogram' with 2 branches and 88 members total, at height 245.04

[[2]]
'dendrogram' with 2 branches and 120 members total, at height 341.3791

[[3]]
'dendrogram' with 2 branches and 225 members total, at height 430.2527
```

```
[[4]]
'dendrogram' with 2 branches and 90 members total, at height 289.0304

[[5]]
'dendrogram' with 2 branches and 168 members total, at height 323.2297

[[6]]
'dendrogram' with 2 branches and 32 members total, at height 151.1788

[[7]]
'dendrogram' with 2 branches and 170 members total, at height 403.9078


inf <- read.delim("CLL_Batches12_Clin_Info_v2_BFRMdata.txt")
a <- labels(cut.D$lower[[1]])
b <- labels(cut.D$lower[[2]])
c <- labels(cut.D$lower[[3]])
d <- labels(cut.D$lower[[4]])
e <- labels(cut.D$lower[[5]])
f <- labels(cut.D$lower[[6]])
g <- labels(cut.D$lower[[7]])

am <- match(a,inf[,1])
bm <- match(b, inf[,1])
cm <- match(c, inf[,1])
dm <- match(d, inf[,1])
em <- match(e, inf[,1])
fm <- match(f, inf[,1])
gm <- match(g, inf[,1])
```

Sub-Sampling of the Combined Dataset:

```
cll.rma <- read.delim("normalized_RMA.gct", skip = 2)
cll.rmadata <- cll.rma[,-c(1:2)]
rownames(cll.rmadata) <- cll.rma[,1]

cols <- dim(cll.rmadata)[2]
x <- as.integer(cols/50)
library(ConsensusClusterPlus)

for(z in 1:25){
        rnd.ord <- sample(1: cols, cols)
        cll.rnd <- cll.rmadata[,rnd.ord]

        for(i in 1:x){
                cll.sub <- cll.rnd[,1:(50*i)]
                cll.sd <- apply(cll.sub, 1, sd)
                cll.sd.ord10perc <- order(cll.sd, decreasing = T)[1:2222]
                cll.sub10perc <- as.matrix(cll.sub[cll.sd.ord10perc,])
                results <- ConsensusClusterPlus(cll.sub10perc, maxK = 8, reps = 50, pItem = .9, pFeature = 1, title
= paste("CLL_CC_", i, "_", z, "rep", sep = ""), clusterAlg = "hc", innerLinkage = "ward", finalLinkage = "ward",
distance = "euclidean", seed = 12345, plot = "png")
                }

        cll.sd <- apply(cll.rnd, 1, sd)
        cll.sd.ord10perc <- order(cll.sd, decreasing = T)[1:2222]
        cll.sub10perc <- as.matrix(cll.rmadata[cll.sd.ord10perc,])
```

```
    results <- ConsensusClusterPlus(cll.sub10perc, maxK = 8, reps = 50, pItem = .9, pFeature = 1, title = paste("CLL_CC_all_", z, "rep", sep = ""), clusterAlg = "hc", innerLinkage = "ward", finalLinkage = "ward", distance = "euclidean", seed = 12345, plot = "png")
    }
```

Evaluation of prognostic markers by group:

```
fish.tbl <- matrix(ncol = 7, nrow = 7)
colnames(fish.tbl) <- names(table(inf[am,4], useNA = "always"))

fish.tbl[1,] <- table(inf[am,4], useNA = "always")
fish.tbl[2,] <- table(inf[bm,4], useNA = "always")
fish.tbl[3,] <- table(inf[cm,4], useNA = "always")
fish.tbl[4,] <- table(inf[dm,4], useNA = "always")
fish.tbl[5,] <- table(inf[em,4], useNA = "always")
fish.tbl[6,] <- table(inf[fm,4], useNA = "always")
fish.tbl[7,] <- table(inf[gm,4], useNA = "always")
chisq.test(fish.tbl[,1:6])

cd38.tbl <- matrix(ncol = 3, nrow = 7)
colnames(cd38.tbl) <- names(table(inf[am,5], exclude = NULL))

cd38.tbl[1,] <- table(inf[am,5], useNA = "always")
cd38.tbl[2,] <- table(inf[bm,5], useNA = "always")
cd38.tbl[3,] <- table(inf[cm,5], useNA = "always")
cd38.tbl[4,] <- table(inf[dm,5], useNA = "always")
cd38.tbl[5,] <- table(inf[em,5], useNA = "always")
cd38.tbl[6,] <- table(inf[fm,5], useNA = "always")
cd38.tbl[7,] <- table(inf[gm,5], useNA = "always")
chisq.test(cd38.tbl[,1:2])

zap.tbl <- matrix(ncol = 3, nrow = 7)
colnames(zap.tbl) <- names(table(inf[am,6], useNA = "always"))

zap.tbl[1,] <- table(inf[am,6], useNA = "always")
zap.tbl[2,] <- table(inf[bm,6], useNA = "always")
zap.tbl[3,] <- table(inf[cm,6], useNA = "always")
zap.tbl[4,] <- table(inf[dm,6], useNA = "always")
zap.tbl[5,] <- table(inf[em,6], useNA = "always")
zap.tbl[6,] <- table(inf[fm,6], useNA = "always")
zap.tbl[7,] <- table(inf[gm,6], useNA = "always")
chisq.test(zap.tbl[,1:2])

mut.tbl <- matrix(ncol = 3, nrow = 7)
colnames(mut.tbl) <- names(table(inf[am,7], useNA = "always"))

mut.tbl[1,] <- table(inf[am,7], useNA = "always")
mut.tbl[2,] <- table(inf[bm,7], useNA = "always")
mut.tbl[3,] <- table(inf[cm,7], useNA = "always")
mut.tbl[4,] <- table(inf[dm,7], useNA = "always")
mut.tbl[5,] <- table(inf[em,7], useNA = "always")
mut.tbl[6,] <- table(inf[fm,7], useNA = "always")
mut.tbl[7,] <- table(inf[gm,7], useNA = "always")
chisq.test(mut.tbl[,1:2])
```

Evaluation of clinical outcome by group:

```
dd.vec <- vector("numeric", 893)
```

```
dd.vec[am] <- 1
dd.vec[bm] <- 2
dd.vec[cm] <- 3
dd.vec[dm] <- 4
dd.vec[em] <- 5
dd.vec[fm] <- 6
dd.vec[gm] <- 7

library(survival)
dd.vec.10137 <- dd.vec[inf[,2] == "10137"]
survdat3 <- read.delim("GSE10137_SurvData.txt")
chisq.test(table(dd.vec.10137, survdat3[,6]))

dd.vec.15490 <- dd.vec[inf[,2] =="15490"]
survdat4 <- read.delim("GSE15490_SurvData.txt")
chisq.test(table(survdat4[,3],dd.vec.15490))

survdat3 <- read.delim("GSE10138_SurvData_3.txt")
dd.vec.10138 <- dd.vec[inf[,2] == "10138"]
survdiff(Surv(survdat3[,7], survdat3[,5])~dd.vec.10138)

dd.vec.10138.2 <- vector("character", 68)
is.na(dd.vec.10138.2) <- T
dd.vec.10138.2[which(dd.vec.10138 == 2)] <- "interferon pathway"
dd.vec.10138.2[which(dd.vec.10138 == 6)] <- "interferon pathway"
dd.vec.10138.2[which(dd.vec.10138 == 5)] <- "receptor signaling"
survdiff(Surv(survdat3[, 7], survdat3[, 5]) ~ dd.vec.10138.2)

gse10138.surv4 <- survfit(Surv(survdat3[, 7], survdat3[, 5]) ~ dd.vec.10138)
pdf("GSE10138_KMOS_By7WardGrps_Updated_010512.pdf")
plot(gse10138.surv4, ylab = "Fraction Alive", xlab = "Time (years)", col = rainbow(7), xlim = c(0,30), lty =
c(1,2,3,1,2,3,1))
legend("bottomright", legend = c("Group 1 (n=7)", "Group 2 (n=1)", "Group 3 (n=26)", "Group 4 (n=14)", "Group 5
(n=11)", "Group 6 (n=7)", "Group 7 (n=2)", "p = 0.004"), col = c(rainbow(7),"white"), bty = "n", lty =
c(1,2,3,1,2,3,1,0))
dev.off()

gse10138.surv5 <- survfit(Surv(survdat3[, 7], survdat3[, 5]) ~ dd.vec.10138.2)
pdf("GSE10138_KMOS_By7WardGrp_IfnvsRcptr_010512.pdf")
plot(gse10138.surv5, ylab = "Fraction Alive", xlab = "Time (years)", col = c("red", "blue"), xlim = c(0,20))
legend("topright", legend = c("Interferon Pathway (n = 8)", "Receptor Signaling (n = 11)", "p = 0.03"), col =
c("red", "blue", "white"), bty = "n", lty = 1)
dev.off()
```

Evaluation of copy number variation by group:

```
cnat <- read.delim("CNAT_Segmented.txt")
ids <- read.delim("GSE16746_IDs.txt", strip.white = T)
dd.vec.16746 <- dd.vec[inf[,2] == "16746"]

gsm <- paste(ids[,1], ".CEL.gz", sep = "")
mt <- match(gsm, as.character(inf[,1]), nomatch = 0)
ids.sub <- ids[1:60,]
ids.sub <- cbind(ids.sub, dd.vec.16746)

write.table(ids.sub, "CLL_CNAT_7WardGrp_010112.txt", quote = F, sep = "\t", row.names = F)
table(dd.vec.16746)/60                    #frequency of each group in this dataset
```

```
del <- read.delim("Deletions_Contingency_010112.txt", row.names = 1)
del[is.na(del)]<-0

chisq.test(x = del[,17], p = c(.35,.02, .13, .05, .05, .05, .35))$p.value
[1] 0.03731894
Warning message:
In chisq.test(x = del[, 17], p = c(0.35, 0.02, 0.13, 0.05, 0.05,  :
  Chi-squared approximation may be incorrect

# rest of the columns were not significant

amp <- read.delim("Amplifications_Contingency_010112.txt", row.names = 1)
amp[is.na(amp)]<-0

chisq.test(x = amp[,1], p = c(.35,.02, .13, .05, .05, .05, .35))$p.value
[1] 0.0004015887
Warning message:
In chisq.test(x = amp[, 1], p = c(0.35, 0.02, 0.13, 0.05, 0.05,  :
  Chi-squared approximation may be incorrect

chisq.test(x = amp[,2], p = c(.35,.02, .13, .05, .05, .05, .35))$p.value
[1] 0.01196015
Warning message:
In chisq.test(x = amp[, 2], p = c(0.35, 0.02, 0.13, 0.05, 0.05,  :
  Chi-squared approximation may be incorrect

# rest of the columns were not significant
```
